# Heparan Sulfate Proteoglycans in Human Colorectal Cancer

**DOI:** 10.1155/2018/8389595

**Published:** 2018-06-20

**Authors:** Carolina Meloni Vicente, Daiana Aparecida da Silva, Priscila Veronica Sartorio, Tiago Donizetti Silva, Sarhan Sydney Saad, Helena Bonciani Nader, Nora Manoukian Forones, Leny Toma

**Affiliations:** ^1^Disciplina de Biologia Molecular, Escola Paulista de Medicina, Universidade Federal de São Paulo, Rua Três de Maio, 100 4° Andar, Vila Clementino, São Paulo, SP, Brazil; ^2^Disciplina de Farmacologia Celular, Escola Paulista de Medicina, Universidade Federal de São Paulo, Rua Três de Maio, 100 4° Andar, Vila Clementino, São Paulo, SP, Brazil; ^3^Disciplina de Gastroenterologia Clínica, Escola Paulista de Medicina, Universidade Federal de São Paulo, Rua Loefgreen 1726, Vila Clementino, São Paulo, SP, Brazil; ^4^Disciplina de Gastroenterologia Cirúrgica, Escola Paulista de Medicina, Universidade Federal de São Paulo, Rua Napoleão de Barros, 715 2° Andar, Vila Clementino, São Paulo, SP, Brazil

## Abstract

Colorectal cancer is the third most common cancer worldwide, accounting for more than 610,000 mortalities every year. Prognosis of patients is highly dependent on the disease stage at diagnosis. Therefore, it is crucial to investigate molecules involved in colorectal cancer tumorigenesis, with possible use as tumor markers. Heparan sulfate proteoglycans are complex molecules present in the cell membrane and extracellular matrix, which play vital roles in cell adhesion, migration, proliferation, and signaling pathways. In colorectal cancer, the cell surface proteoglycan syndecan-2 is upregulated and increases cell migration. Moreover, expression of syndecan-1 and syndecan-4, generally antitumor molecules, is reduced. Levels of glypicans and perlecan are also altered in colorectal cancer; however, their role in tumor progression is not fully understood. In addition, studies have reported increased heparan sulfate remodeling enzymes, as the endosulfatases. Therefore, heparan sulfate proteoglycans are candidate molecules to clarify colorectal cancer tumorigenesis, as well as important targets to therapy and diagnosis.

## 1. Background

Colorectal cancers (CRC) arise from the epithelium lining the colon or rectum. In females and males, it is the third and fourth most common cancer, respectively. This type of cancer is responsible for 610,000 mortalities worldwide yearly [1]. The incidence of CRC tends to increase considering aging and population growth [2]. The survival rate of patients with CRC is hugely dependent on the disease stage and in a projected five-year survival rate; patients with stage I tumors show range from 85 to 90% while the range is less than 5% for patients with stage IV diseases [3]. The main risk factors are the following: age over 50 years; family history of colon and rectal cancer, including some hereditary conditions (family adenomatous polyposis (FAP) and hereditary nonpolyposis colorectal cancer (HNPCC)); high-fat content in diet, meat consumption, and low calcium content; physical inactivity and obesity; and inflammatory colon diseases such as chronic ulcerative colitis and Crohn's disease [4, 5].

CRC is commonly diagnosed in advanced stages in both sexes and presents a higher incidence after 55 years of age. CRC screening methods increase the early diagnosis of this pathology and allow the identification of premalignant lesions such as adenomatous polyps [6, 7]. In addition to colonoscopy, rectosigmoidoscopy and occult blood decrease CRC mortality as screening methods. Thus, when people are screened in their fifties and the polyps are removed, the subsequent incidence of colorectal cancer is usually very low [8, 9].

Therefore, CRC is curable in nearly 90% cases if detected at an early stage. Furthermore, screening methods detecting mucosal changes reduce the incidence and mortality rates of this disease [10]. The most accurate method of diagnosis is colonoscopy, followed by histopathological biopsy. Fecal occult blood test (FOBT) is the most noninvasive screening procedure used and is able to reduce CRC-related mortality by 20%, when executed every other year [11]. In spite of improvements in sensitivity, FOBT has a low detection rate for early-stage tumors and precancerous lesions, such as polyps [1, 12]. Even though colonoscopy and rectosigmoidoscopy are more effective in detecting CRC, they are extremely costly and require extensive preparation of the bowel and involve invasion of patient privacy and sedation [13]. As a rule, surgery is the primary treatment, removing the affected portion of the intestine and lymph nodes near this region. After surgical procedure, chemotherapy or radiotherapy can be recommended in order to reduce the tumor recurrence [14].

In 1990, Fearon and Vogelstein suggested a model for colorectal cancer tumorigenesis, which describes the genetic alterations involved in transformation from normal intestinal mucosa to colorectal carcinoma [15]. Thenceforward, CRC critical genes have already been well established, 40% of the cases of CRC have a specific point mutation in KRAS, 60% have inactivating mutations or deletions of p53, and more than 60% have mutations in the APC (adenomatous polyposis coli) tumor suppressor gene. Additional studies have revealed how these genes lead to uncontrolled cell division and metastasis [16, 17].

The inactivation of the APC gene appears to be a very early step in most CRC cases, since it can be detected already in small benign polyps at the same high frequency as in malignant tumors. Loss of APC function appears to be responsible for the increase of cell proliferation [18]. Mutations involving the KRAS oncogene appear to take place later than those in APC as they are infrequent in small polyps but common in larger ones that present undifferentiated cells [19]. Finally, mutations in p53 are rare in polyps but common in carcinomas, suggesting that they may often occur late in the sequence. Loss of p53 function leads abnormal cells to avert apoptosis, divide, and promote the accumulation of additional mutations [20].

Not only genetic mutations and chromosome instability but also another frequent genomic instability in CRC is the microsatellite instability at the nucleotide level, commonly resulting in deletions or insertions of a few nucleotides [21, 22]. Furthermore, global DNA hypomethylation and depletion of overall 5-methylcytosine content in CRC tissues were observed for the first time in 1983, by Feinberg and Vogelstein [23]. This global hypomethylation has been associated with an increased genomic instability and overexpression of genes implicated in CRC pathogenesis [24]. Moreover, this hypomethylation is believed to be associated with the hypermethylation at the promoter regions of specific genes that are involved in cell cycle regulation, DNA repair, apoptosis, angiogenesis, adhesion, and invasion [1, 25].

**Figure 1:**
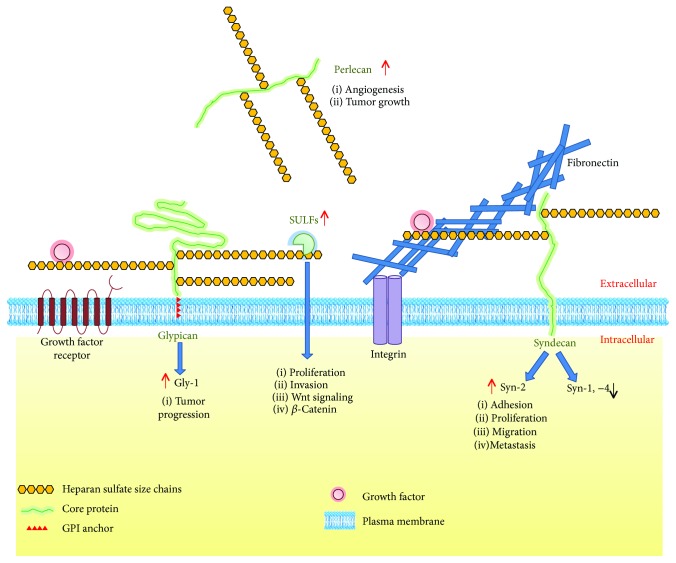
Putative model of the functions of HSPGs in CRC cells. The cell surface HSPG syndecan-2 (Syn-2) is upregulated and promotes cancer cell adhesion, proliferation, migration, and metastasis. Syndecan-1 and syndecan-4 (Syn-1; Syn-4), generally antitumor molecules, are reduced in colon carcinoma cells. The cell surface HSPG glypican-1 (Gly-1) is increased in CRC and is involved in tumor progression. The augmentation of matrix HSPG perlecan favors angiogenesis and tumor growth. The SULF enzymes are upregulated, and the edition of HS chains promotes proliferation and invasion of CRC cells. In addition, SULFs release growth factors that were bound to HS, stimulating the Wnt signaling pathway and the activation of *β*-catenin.

It has been known for decades that proteoglycans (PG) are involved in the progression of cancer at various stages. Heparan sulfate proteoglycans (HSPGs) play vital roles in tumorigenesis, allowing cancer cells to proliferate, evade immune response, invade adjacent tissues, and metastasize to distal sites away from the primary tumor [26]. In CRC, syndecan-1 and syndecan-4 are downregulated while syndecan-2 is upregulated [27–29] (Figure 1). In addition, studies have reported increased 6-OST, heparanase [30, 31], and SULFs [32, 33]. Notably, several of CRC critical genes show relationship with HSPGs. For instance, p53 has been described to regulate the expression of SULF2 or heparanase. Many growth factors, including TGF-beta and VEGF, bind to heparan sulfate chains; the WNT/beta-catenin pathway is regulated by glypicans and SULFs [34, 35].

## 2. Heparan Sulfate Proteoglycans

HSPGs are complex molecules presenting one or more heparan sulfate (HS) chains covalently bound to the protein backbone [36], being present on the cell surface and extracellular matrix (ECM) of all animals with tissue organization [37–41]. They can be distributed into three groups, depending on their cellular localization: membrane HSPGs (as syndecans and glypicans), HSPGs secreted into the ECM (perlecan, collagen-type XVIII), and the HSPG serglycin that is located in cell vesicles [42, 43].

The biological functions of HSPGs are very varied, and there is no common denominator. Many of their functions depend on the interaction with the protein backbone, while others depend on sugar chains [44, 45]. Among many roles, the HSPGs are present in basement membranes, where they collaborate with other matrix components to define their structure and assist in cell migration [46]. They are also found in secretory vesicles (serglycin) participating in the granular content packaging, activation of proteases, and regulating activities after secretion such as coagulation and wound healing [47].

At the cell surface, the HSPGs may bind to cytokines, chemokines, and growth factors; in this way, these PGs protect themselves from proteolysis or act as coreceptors [48]. These interactions provide a deposit of regulatory factors that can be released by selective degradation of HS chains. Acting as receptors for proteases or protease inhibitors, HSPGs regulate their spatial distribution and activity [49]. Membrane HSPGs may cooperate with different cell adhesion receptors such as integrins and facilitate cell-ECM adhesion, cell-cell interactions, and cell motility [50, 51].

Therefore, several cellular mechanisms regulated by HSPG are critically involved in cancer. There is an abundance of evidence relating HSPG fine structures to cancer growth, invasion, and metastasis. Through the aberrant modulation of HS biosynthetic enzymes, the specific HS fine structure enables cancer cells to spread by the breakdown of ECM, to receive nutrients through angiogenesis, to proliferate via disruption of signaling pathways, and to escape immune cells. In addition, different levels of HSPG core proteins are involved in several tumor-promoting processes [26, 52].

### 2.1. Cell Surface HSPG

#### 2.1.1. Syndecans

The syndecans are a family of four transmembrane proteoglycans that bear predominantly heparan sulfate glycosaminoglycan chains [28]. The core proteins consist of a short intracellular domain, a highly conserved transmembrane domain, and an ectodomain that is divergent in amino acid sequence among the four syndecan family members [41].

The syndecans regulate cell adhesion, migration, cytoskeleton organization, and gene expression through the binding of ECM molecules and soluble ligands [40]. Since cancer cells exhibit less adhesive and more migratory characteristics in comparison to normal cells, syndecans are candidate molecules to be differently regulated in cancer cells. Therefore, it is probable that syndecans may influence cell morphology, adhesion to the ECM, and tumorigenic activity.

**Figure 2:**
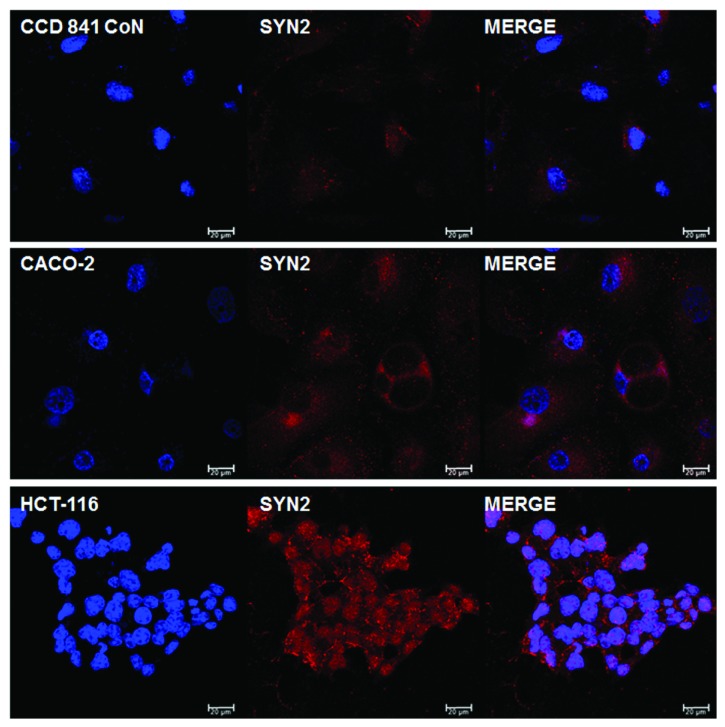
Expression of syndecan-2 (Syn-2) in normal colorectal cell line (CCD 841 CoN), in nonmetastatic CRC cell line CACO-2, and in high metastatic CRC cell line HCT-116. Immunostaining (red) was detected using an antibody specific for syndecan-2 (Santa Cruz) and an Alexa Fluor 594-labeled secondary antibody. Cell nuclei were stained with DAPI (blue). Images were obtained using a confocal a microscope Leica Microsystems TCS SP8 and analyzed by software LAS-AF.

According to the literature, syndecan-2 is the most involved in CRC. Syndecan-2 regulates cell adhesion in several cell lines including epithelial cells [53], neuronal cells [54], and mesenchymal cells [55]. Moreover, different reports indicate that syndecan-2 positively regulates cell migration, since it is highly expressed in cells under migratory conditions [56]. Park et al. [57] demonstrated that syndecan-2 mRNA levels were increased in CRC cell lines compared with a normal colon cell line. Our results corroborate with these data (Figure 2). The addition of purified recombinant extracellular domain of syndecan-2 to the cell medium completely blocked the adhesion of colon cancer cells on the ECM. Moreover, it induced G0/G1 cell cycle arrest with concomitant increase in p21, p27, and p53 expressions. Therefore, in CRC, syndecan-2 plays a critical role in adhesion of colon carcinoma cells onto the ECM, regulating the proliferation and tumorigenic activity in colon carcinoma cells [57].

It has been well established that the extracellular domain of syndecan-2 interacts with fibronectin [58]. In CRC, the contact between cancer cells with fibronectin enhances syndecan-2 expression, promoting a migratory behavior of highly metastatic tumor cells [29]. In addition, HCT116 transfected with syndecan-2 presented increased cell migration, which was diminished by the knockdown of integrin alpha2 using a specific siRNA [59]. Therefore, this dynamic interaction, including syndecan-2, fibronectin, and integrin, might be a possible mechanism underlying the metastatic characteristics of colon cancer cells. In addition, Choi et al. [60] reported that the overexpression of syndecan-2 enhanced migration and invasion of Caco-2 and HCT116 cells through Tiam1-mediated activation of Rac, a GTPase family member involved in cell contact regulation.

Finally, it has been recently reported that in HT29 cells, syndecan-2 overexpression promotes E-cadherin shedding to the conditioned medium [61]. Consistently, the overexpression of syndecan-2 in HT29 cells increased the expression and secretion of MMP-7 whereas siRNA-mediated knockdown of MMP-7 in these same cells significantly increased E-cadherin levels. The shedding of E-cadherin disrupts cell-cell adhesion and induces cells to undergo morphological changes toward a fibroblast-like phenotype, inducing the epithelial-mesenchymal transition in CRC cells.

On the other hand, syndecan-1 has been associated with a tumor suppressor function [62]. Similarly, syndecan-4, which is mainly involved in cytoskeletal and membrane reorganization and formation of focal adhesions, inhibits cell migration and tumor activity [63]. Consistently, mRNA expression of syndecan-1 and syndecan-4 is significantly reduced in colon carcinoma cells [40]. However, in different types of cancers, syndecan-1 and syndecan-4 may present the opposite effect, promoting the tumor progression [64, 65]. In addition, it has already been demonstrated that the shed of syndecan-1 is associated with chemotherapy resistance in castration-resistant prostate cancer [66]. These data evidence that the function of cell surface HSPGs can be altered by extracellular ectodomain shedding by proteases, converting them into soluble paracrine effector molecules. It is worth mentioning that the shedding of HSPGs is a controlled mechanism that can occur constitutively and can be substantially enhanced by exogenous stimuli or by a pathogenic state, including cancer [67].

#### 2.1.2. Glypicans

Glypicans (GPCs) constitute a family of HSPGs externally linked to the plasma membrane by a glycosylphosphatidylinositol (GPI) anchor [68]. In mammals, the glypican family comprises six members, GPC1 to GPC6. GPCs can modify cell signaling pathways including Wnts, hedgehogs, fibroblast growth factors, and bone morphogenetic proteins, which are mainly involved in cellular proliferation and tissue growth [68]. GPC functions may either be stimulatory or inhibitory through these different pathways.

GPC1 has been implicated in tumor progression events, such as growth, angiogenesis, and metastasis, and has been especially studied in pancreatic cancer, glioma, and breast cancer [69]. De Robertis et al. [70] found that the GPC1 gene was significantly upregulated in azoxymethane/dextran sodium sulfate (AOM/DSS) mouse model, which mimics human CRC. Results were confirmed by immunohistochemical analysis in 10 human tumor cases and 10 normal matched mucosa specimens, revealing a strong increase of membrane/cytoplasmic staining for GPC1 in 80% of tumors.

Several studies have demonstrated a correlation between GPC3 expression levels and various types of cancer. Downregulation of GPC3 has already been detected in ovarian carcinoma, breast cancer, and mesothelioma, suggesting that it may act as a tumor suppressor gene in these tissues [67]. In contrast, GPC3 is upregulated in hepatocellular carcinoma, germ cell tumor, and lung squamous cell carcinoma, suggesting that GPC3 may also behave as an oncofetal protein [68].

In CRC, downregulation of GPC3 mRNA levels was observed in all 10 tumor samples, compared to normal mucosa [70]. Moreover, a retrospective study involving 150 CRC cases reported that nonmucinous carcinoma (NMA) showed a higher expression of GPC3 than did mucinous carcinoma (MA), which is associated with worse prognosis [71]. Interestingly, GPC3 immunohistochemistry analysis demonstrated a strong staining in normal mucosa and a cytoplasmic staining in tumor cells.

### 2.2. Matrix HSPG

#### 2.2.1. Perlecan

Having a large multidomain, perlecan is a proteoglycan of five domains secreted to the extracellular matrix. It has homology to growth factors, immunoglobulin, and adhesion molecules [69]. Perlecan is able not only to bind but also to cross-link many ECM components and cell-surface molecules. By collaborating with other matrix components, perlecan defines the basement membrane structure and provides a matrix for cell migration [72]. Moreover, it was discovered that perlecan exhibits high-affinity binding of fibroblast growth factor- (FGF-) 2, a proangiogenic factor, to cells lacking heparan sulfate and to the FGF receptor [69].

Perlecan is an important component of the vascular ECM. Different studies have suggested that perlecan could function as an initial scaffold upon which endothelial cells would migrate and deposit an appropriate vascular basement membrane [38, 39]. Several independent studies using antisense RNA strategies in various tumor cells have confirmed the central role of perlecan in angiogenesis, with both *in vitro* and *in vivo* models [38].

Perlecan suppression caused significant tumor reduction and inhibition of angiogenesis in human CRC tumor xenografts [73]. Proliferation of HCT116 human CRC cells was markedly reduced upon obliteration of perlecan gene expression by an antisense cDNA, and these effects correlated with reduced responsiveness to FGF-7.

Interestingly, perlecan was more expressed in the AG2 colon cancer-initiating cell line, compared to the carcinoma cells HCT116. However, the gene expression of perlecan was downregulated 2-fold in colon tumors from 12 patients, using the surrounding tissue as control [74]. Therefore, the function of perlecan in CRC requires to be better clarified.

## 3. HSPG Biosynthetic Enzymes

In general terms, the initial HS chain is synthesized by the alternating action of different glycosyltransferases, which add D-glucuronic acid (GlcA) and N-acetyl-D-glucosamine (GlcNAc) residues. Subsequently, the chain undergoes a series of reactions of polymer modifications: N-deacetylation/N-sulfation, epimerization of *β*-D-glucuronic acid residue to *α*-L-iduronic acid, and O-sulfation in different positions [75]. Each product of one reaction is a substrate for the next enzyme [76], and 3′-phosphoadenosine-5′-phosphosulfate (PAPS) is used as a sulfate donor by sulfotransferases [77]. The length of the HS chain, as well as its degree of sulfation, may vary depending on the protein skeleton and the cell type [76].

On the cell surface or ECM, two endosulfatases (SULF1 and SULF2) can further modify the HS chains by removing specific C6-located sulfate groups from the glucosamine units or by the action of extracellular heparanase or proteases [77].

### 3.1. SULFs

Being located on the cell surface or released into the ECM, SULFs represent a family of secreted enzymes that selectively remove 6-O-sulfate groups from HS, with preference for those present in trisulfated disaccharides [78].

After cloning the human SULF cDNA, analyses of SAGE databases provided the first indication that these genes are relevant for cancer. SULF1 and SULF2 occur with a higher frequency in three types of human tumors (breast, central nervous system, and colon) compared to normal tissues [79, 80].

In more recent studies, the overexpression of SULFs in a wide range of tumors has been reported through quantitative PCR or gene microarray: SULF1 is upregulated in hepatocellular carcinoma [81], gastric cancer [82], head and neck carcinoma [83], pancreatic cancer [84], and lung adenocarcinoma [85], and SULF2 is highly expressed in hepatocellular carcinomas [86] and lung carcinoma [85], among others.

Stable overexpression of SULFs in the CRC cells, Caco-2, and HCT-116 induced an increase in cell viability and proliferation and augmented cell migration [33]. These effects were reversed by shRNA-mediated knockdown of SULF1 or SULF2 and by the addition of unfractionated heparin to the cell medium. Moreover, CRC cell lines overexpressing SULFs presented increased Wnt signaling, represented by the accumulation of active nonphosphorylated beta-catenin in the cells. Ai et al. [87] proposed a model by which SULFs could promote Wnt signaling. The model suggests that the action of SULFs weakens the association of Wnt ligands with HSPGs on the cell surface, which allows ligands to activate signal transduction receptors (frizzled).

**Figure 3:**
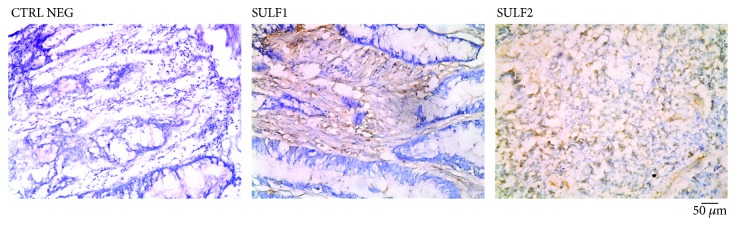
Expression of SULF1 and SULF2 in CRC tissue sample. Immunostaining was detected using an antibody specific for SULF1 or SULF2 (Santa Cruz) and HRP peroxidase/DAB reaction. Tissue samples were stained after with hematoxylin. Images were obtained using a Nikon Eclipse microscope.

In addition, the gene expression of SULFs in human CRC tissue samples revealed a significant increase of those sulfatases, which argues for a possible distortion of HS sulfation patterns in colon tumors [74] (Figure 3). Therefore, these studies reveal that SULFs have oncogenic effects in CRC, suggesting an important role for these enzymes in cancer progression.

## 4. Conclusions and Clinical Relevance

Dysregulated expression of HSPGs, as well as of enzymes involved in their biosynthesis and degradation, has been reported to affect all stages of tumorigenesis [88]. As extracellular proteins, HSPGs and the extracellular enzymes that modify them, such as SULFs, are amenable to therapeutic targeting [89]. Heparan sulfate mimetics, highly sulfated oligosaccharides, inhibit SULF functions and sequester HS-binding ligands, making them attractive candidates for cancer therapy [90, 91]. It is noteworthy that an inhibitor of SULFs has already been identified, named PI-88 [92]. This agent consists of a mixture of chemically sulfated yeast oligosaccharides with a molecular weight range of 1400–3100 Da. This compound has been tested in clinical trials for advanced melanoma (phase II), liver cancer, lung cancer, and prostate cancer. However, these studies have demonstrated recurring problems of immune-mediated thrombocytopenia in a significant number of patients associated with the use of PI-88 [93]. Therefore, both the detection and the inhibition of SULFs can present clinical value for CRC treatment.

As demonstrated, syndecan-2 is a candidate for CRC diagnosis. Shed or secreted proteoglycans and their extracellular modifying enzymes can often be detected in the blood [94]. As these are often altered in cancer, changes in their blood levels may be useful as biomarkers of disease. Moreover, the inhibition of syndecan-2 could reduce tumor cell migration, protecting CRC patients from metastasis.

In addition to potential direct antitumor effects, therapeutic targeting of HSPGs in CRC could also modulate angiogenesis. The inhibition of perlecan in early stages of CRC could contribute to preventing tumor development. Therefore, these studies illustrate the critical importance of HSPGs in all stages of CRC and reinforce the relevance of conducting preclinical studies to test the therapeutic efficacy and safety of potential targeting agents.

Based on these important functions, the question arises as to whether HSPGs can be utilized as potential candidate molecules for CRC diagnosis and treatment. First, as mainly extracellular molecules, they can be easily achieved by different mechanisms, being interest targets for cancer therapy, which could include the usage of specific antibodies targeting HSPGs. In addition, the detection of the HSPG ectodomain or SULF levels in the serum or stool samples emerge as promising diagnostic tools for CRC patients.

Furthermore, HSPGs are involved in all tumor stages, including cell proliferation and migration, metastasis, and angiogenesis. Therefore, it is worth exploring the still unknown complex molecular events involving HSPGs. However, appropriate studies are crucial to deciphering the paradoxes of the involvement of different isoforms of HSPGs in CRC. Finally, a highly promising next step will be the development of precise inhibitors for specific types of HSPG, which would contribute to a better comprehension of the roles of HSPGs in CRC. This may represent the greatest challenge since HSPGs have different isoforms and possess ambiguous roles. However, the development of these molecules could represent an important step towards the application of HSPGs in clinical trials.

## Abbreviations

PAPS: 3′-Phosphoadenosine-5′-phosphosulfate
APC: Adenomatous polyposis coli
CRC: Colorectal cancers
GlcA: D-Glucuronic acid
ECM: Extracellular matrix
FAP: Family adenomatous polyposis
FOBT: Fecal occult blood test
(FGF)-2: Fibroblast growth factor
GPI: Glycosylphosphatidylinositol
GPCs: Glypicans
HS: Heparan sulfate
HNPCC: Hereditary nonpolyposis colorectal cancer
MMP: Metalloproteinase
MA: Mucinous carcinoma
GlcNAc: N-Acetyl-D-glucosamine
NMA: Nonmucinous carcinoma
PCR: Polymerase chain reaction
PG: Proteoglycans
SULF: Endosulfatase
TGF-beta: Transforming growth factor
VEGF: Vascular endothelial growth factor.
